# Effects of minerals and xylitol solution on human nasal epithelium: Water channels upregulation and potential anti-inflammatory properties

**DOI:** 10.1016/j.bjorl.2026.101889

**Published:** 2026-09-02

**Authors:** Michelle Sabrina da Silva, Ana Laura Gaspar, Douglas Costa Morais, Fábio Ikedo, Flávia Cardoso Males, Gabriela Pacheco de Oliveira, Mariana Rodrigues Pinto, Rodrigo Torres Scabello, Stevin Zung, Edwin Tamashiro

**Affiliations:** a MEDCINVITRO – Medcin Group, Brazil; b Aché Laboratórios Farmacêuticos S.A., Brazil; c Universidade de São Paulo, São Paulo, SP, Brazil

**Keywords:** Nasal epithelium, Xylitol, Minerals, Aquaporin-3, Anti-inflammatory properties

## Abstract

**Objectives:**

The nasal mucosa has a fundamental role in defending the respiratory system by filtering inhaled air, maintaining hydration, and regulating osmotic balance. This study aimed to evaluate the safety and potential benefits of an Investigational Product (IP), composed of saline solution 0.9%, minerals and xylitol 1%, on nasal epithelial cells using a reconstructed nasal epithelium model *in vitro*.

**Methods:**

The IP’s impact on key mediators such as Aquaporin-3 (AQP3), Adenosine Triphosphate (ATP), and Interleukin-8 (IL-8) synthesis was assessed. For comparison, specific positive controls were used for each cell viability and epithelium integrity assays, while saline solution 0.9% was applied as the negative control.

**Results:**

Treatment with the IP resulted in a 64% increase in AQP3 expression, suggesting enhanced cellular hydration and osmotic balance. Moreover, the IP reduced ATP release by 36% on day-1 and 40% on day-4, indicating its potential to modulate epithelial responses to stress. Inflammatory cytokine IL-8 synthesis also decreased by 25% and 31% on days-1 and -4, respectively, when compared to the inflammatory induced group, supporting the IP’s anti-inflammatory properties potential. Importantly, the IP did not induce cytotoxicity or irritative responses, maintaining cellular viability and integrity.

**Conclusion:**

These results suggest that the IP could be a promising therapeutic option for modulating inflammation and maintaining nasal epithelial function in inflammatory conditions, such as rhinitis and rhinosinusitis. Further *in vivo* studies and clinical trials are warranted to confirm these findings and to explore the therapeutic applicability of this product in nasal health management.

**Level of evidence:**

Level 5.

## Highlights

• Xylitol-Mineral solution (XTM) is safe to the human nasal epithelium.
• XTM does not induce pro- and inflammatory cytokines expression.
• XTM preserves mucociliary transport.
• XTM enhances aquaporin-3 expression, supporting potential epithelial hydration.
• XTM downregulates inflammatory responses under LPS/TNF-alfa stimulation.

## Introduction

Nasal mucosa is a pseudostratified tissue that filters inhaled air and provides defense against foreign agents, in addition to allowing the colonization of commensal microbiota.^1^, ^2^, ^3^ Under physiological conditions, the mucosa of the nose and paranasal sinuses produces approximately 600–1800 mL of mucus daily in adults, primarily secreted by goblet cells and glands present in the stroma. Although the composition of mucus varies among individuals, it consists mostly of water (∼95%), along with proteins, peptides (2%–3%), mineral salts (1%), and cellular debris (1%). Mucus forms a protective layer over the respiratory mucosa, trapping and clearing pollutants, allergens, and infectious agents toward the digestive tract through the synchronized ciliary movement of the respiratory epithelium.^4^

Dysfunction of the epithelial barrier is a key starting point process in triggering and maintaining inflammatory responses in the nasal mucosa. Increased permeability of the nasal mucosa exposes innate immune cells and trigeminal receptors to noxious environment particles, inducing the recruitment of inflammatory cells, production of pro-inflammatory cytokines, mucous production, and stimulation of reflexes such as sneezing and itching.^2^ The nasal epithelial barrier integrity status is closely linked to allergic disease pathology, such as allergic rhinitis and chronic rhinosinusitis, and can therefore be a target for therapeutic approaches.^5^

The use of saline solutions for nasal hygiene is a well-established practice for patients with allergic and non-allergic rhinitis, acute and chronic rhinosinusitis, and even non-specific conditions such as the presence of postnasal drip. Among the various benefits reported in the literature, nasal lavage provides mechanical cleansing of crust and debris, reduces the viscosity of mucus, stimulates ciliary beat frequency, and optimizes mucociliary clearance.^6^

In addition to sodium chloride, other ingredients have been tested to potentiate the effects of nasal lavage. Xylitol, a 5-carbon sugar alcohol, has been associated with improvement in nasal conditions when associated with nasal irrigation. Xylitol increases nasal Nitric Oxide (NO) levels and inducible Nitric Oxide Synthase (iNOS) mRNA expression in the maxillary sinus, which may contribute to its therapeutic effects in chronic rhinosinusitis.^7^ In the experimental rat model, xylitol showed histopathological improvements in the nasal mucosa in treatment of drug-induced rhinitis, a condition caused by prolonged use of nasal decongestants.^8^ Xylitol nasal irrigation has also been linked to symptom improvement in chronic rhinosinusitis, indicating better sinonasal function compared to saline irrigation.^9^ Additionally, xylitol may enhance bacterial killing by lowering the salt concentration of airway surface liquid, improving innate antimicrobial defenses. This effect has been observed both *in vitro* and *in vivo*, including reduced nasal bacterial carriage and potential benefits in managing sinusitis.^10^

Other additives that are less explored in nasal lavage are minerals. Minerals play an important role in the homeostasis and metabolic regulation of the respiratory epithelium. In the nasal mucus, electrolytes such as sodium, potassium, calcium, magnesium, and chloride influence processes such as ciliary beating and epithelial cell secretion.^4^ Chloride, for example, acts in osmotic balance and mucus viscosity,^11^ zinc can modulate ciliary activity through the activation of purinergic receptors,^12^ while potassium participates in ion transport and mucus hydration.^13^ Additionally, magnesium plays a relevant role in modulating inflammatory responses and oxidative stress, influencing pathways related to inflammation.^14^^,^^15^ Our hypothesis is whether the combination of xylitol and minerals in nasal lavage may provide additional benefits in physiological and inflammatory conditions.

The objective of this study is to evaluate the safety and potential benefits for nasal mucosa of an Investigational Product (IP), composed of saline solution 0.9%, xylitol 1%, and minerals, using an *in vivo* reconstructed nasal epithelium model.

## Methods

### Reconstructed nasal epithelium model and treatment protocol

Reconstruction of the nasal epithelium was performed using RPMI 2650 cells (BCRJ), seeded in culture flasks using EMEM (Sigma) culture medium with 10% fetal bovine serum and 1% gentamicin and incubated at 37 °C with 5% CO_2_ until reaching 95% of confluence, as described by Wengst et al.^16^ with adaptations. The cells were seeded in Transwell inserts with a 0.4 μm pore size at a density of 2 × 10^5^/cm^2^ and maintained in a liquid-liquid interface for 7-days, with the culture medium replaced every 2-days. On the 8th experimental day, the cells were transitioned to an air-liquid interface by removing the culture medium from the apical surface, until the 20th for complete differentiation. After differentiation, the tissues were treated with IP, negative control (saline 0.9%), and positive controls, all at a concentration of 30 μL/cm^2^, once a day for 4 days. The following solutions were used as positive controls: 0.1% Triton X for cytotoxicity evaluations (MTT and LDH), irritant potential assessment (IL-1a) and barrier preservation measurement (Transepithelial Electrical Resistance ‒ TEER); hypotonic solution (1 mM CaCl_2_ and 1 mM MgCl_2_) for ATP measurement; inflammatory mix solution (1% serum, 0.2 mg/mL lipopolysaccharide, and 500 ng/mL TNF-α) for anti-Inflammatory response (IL-8). The reconstructed tissues and supernatants were collected daily for subsequent quantification of biological markers. At the end of the experiments, a Mycoplasma detection test was performed using luminescence, to confirm the absence of Mycoplasma in the cultures (data not shown).

IP is composed of purified water, sodium chloride 0.9%, xylitol, and minerals (magnesium chloride, monobasic sodium phosphate, sodium hydroxide, potassium chloride, and zinc chloride).

### Cell viability, cytotoxic potential and irritative potential

After treatment with IP, the cultures were collected daily for subsequent cellular viability quantification using the MTT (3-(4, 5-dimethylthiazolyl-2)-2, 5-diphenyltetrazolium bromide) salt reduction colorimetric assay technique. The epithelia were incubated with 1.3 mL of MTT (3-(4,5-dimethylthiazol-2-yl)-2,5-diphenyltetrazolium bromide – 1 mg/mL) for 3 -hs at 37 °C. Subsequently, formazan extraction was performed using 1.5 mL of isopropanol, maintained under agitation at room temperature for 2-h, followed by optical density reading at 570 nm.

For the cytotoxic evaluation and irritative potential, the cultures were collected daily. The quantification of Lactate Dehydrogenase (LDH ‒ Elabioscience) and Interleukin-1 alpha (IL-1α ‒ R&D Systems kit), in the culture supernatants, was performed using the Enzyme-Linked Immunosorbent Assay (ELISA) technique.

All tests were conducted using saline solution as a negative control, and 0.1% Triton X-100 as a positive control for damage.

### Transepithelial electrical resistance measurement – TEER

To assess the barrier integrity of the reconstructed nasal epithelium after treatment with IP, TEER measurements were performed, following methodology adapted from Sibinovska et al.,^17^ with a voltohmmeter (MILLICELL-ERS) for 5 consecutive days: before the first treatment, and daily after 24-h of each treatment day. The measurement was performed at an angle of 90° in relation to the inserts and the values were expressed in Ω. An insert without cells was used as a blank.

### Aquaporin-3 expression

The reconstructed epithelia were fixed in 4% paraformaldehyde (pH = 7.4) for 24-h on days-1 and -4 of treatment. Serial sections of 10 μm were then collected directly onto silanized slides using a cryostat. The sections were submitted to three 5-min washes with PBS (Phosphate-Buffered Saline) and were incubated overnight with the primary antibody anti-Aquaporin-3 (1:100 ‒ Novus Biological). Subsequently, the sections were subjected to new PBS washes and incubated for 1 -h with the secondary antibody (1:500 – Life Technologies). Immediately after this step, a 5-minute incubation with DAPI (4′,6-diamidino-2-phenylindole, a fluorescent stain for DNA ‒ 1:5000 ‒ Sigma) was performed, followed by three additional 5-minute washes with PBS. After immunofluorescence procedures, the slides were mounted with a specific mounting medium, analyzed, and photographed using a fluorescence microscope. Following image acquisition, fluorescence intensity was quantified using ImageJ software (National Institutes of Health, USA) with Arbitrary Units (A.U.)

### ATP quantification

The culture supernatants were collected on days-1 and -4 of treatment for subsequent Adenosine Triphosphate (ATP) quantification using the Enzyme-Linked Immunosorbent Assay (ELISA) technique (Cloud-Clone kit). Saline solution was used as a negative control, and a hypotonic solution (1 mM CaCl_2_ and 1 mM MgCl_2_) was used as a positive control.

### IL-8 quantification

The culture supernatants were collected on days-1 and -4 of treatment for subsequent Interleukin-8 (IL-8) quantification using the Enzyme-Linked Immunosorbent Assay (ELISA) technique (R&D Systems kit). Saline solution was used as a negative control, and an inflammatory mix solution (1% serum, 0.2 mg/mL lipopolysaccharide, and 500 ng/mL TNF-α) was used as a positive control.

### Statistical analysis

For statistical evaluation, a non-parametric ANOVA (Analysis of Variance) was used, followed by the Bonferroni post hoc test. A 5% significance level was applied (GraphPad Prism v9).

## Results

### Cytotoxic and irritative potential

**Fig. 1:**
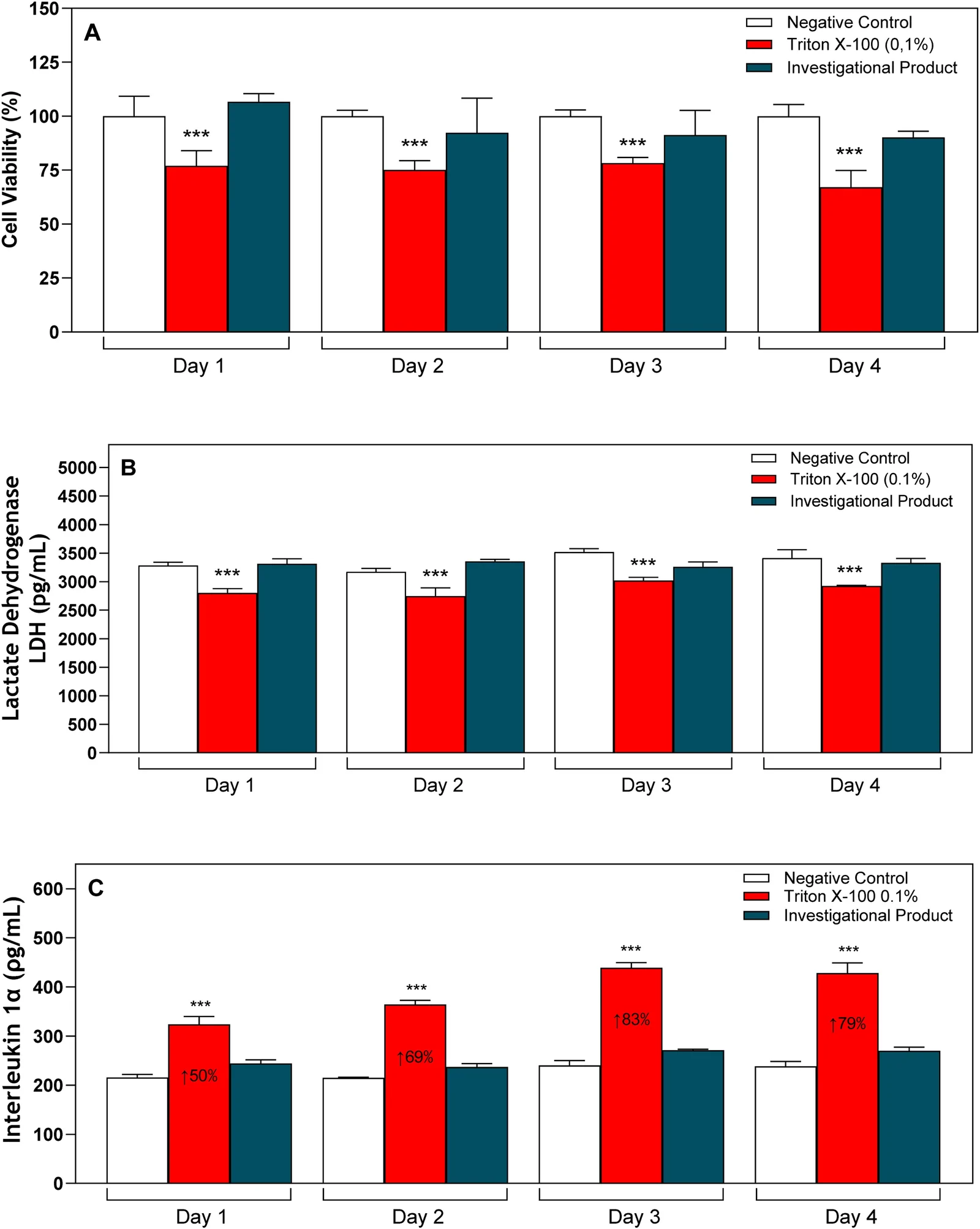
Cytotoxic and irritative potential of the investigational product in a reconstructed nasal epithelium model. The epithelia were treated with IP, negative, and positive control during 4-days for subsequent evaluations. (A) Cell viability by MTT measurement. (B) LDH quantification. (C) IL-1α quantification. *** *p* < 0.001 in relation to the respective negative control of each day.

Exposure of the reconstructed nasal epithelia to positive control (Triton X-100 ‒ 0.1%) led to a statistically significant reduction in cell viability when compared to the respective negative controls. On the other hand, treatment of the reconstructed nasal epithelia with the IP maintained cell viability similar to that observed in the negative control group on all evaluated days (Fig. 1A). These results show that the IP does not induce the cell death process, maintaining tissue viability standards.

**Fig. 2:**
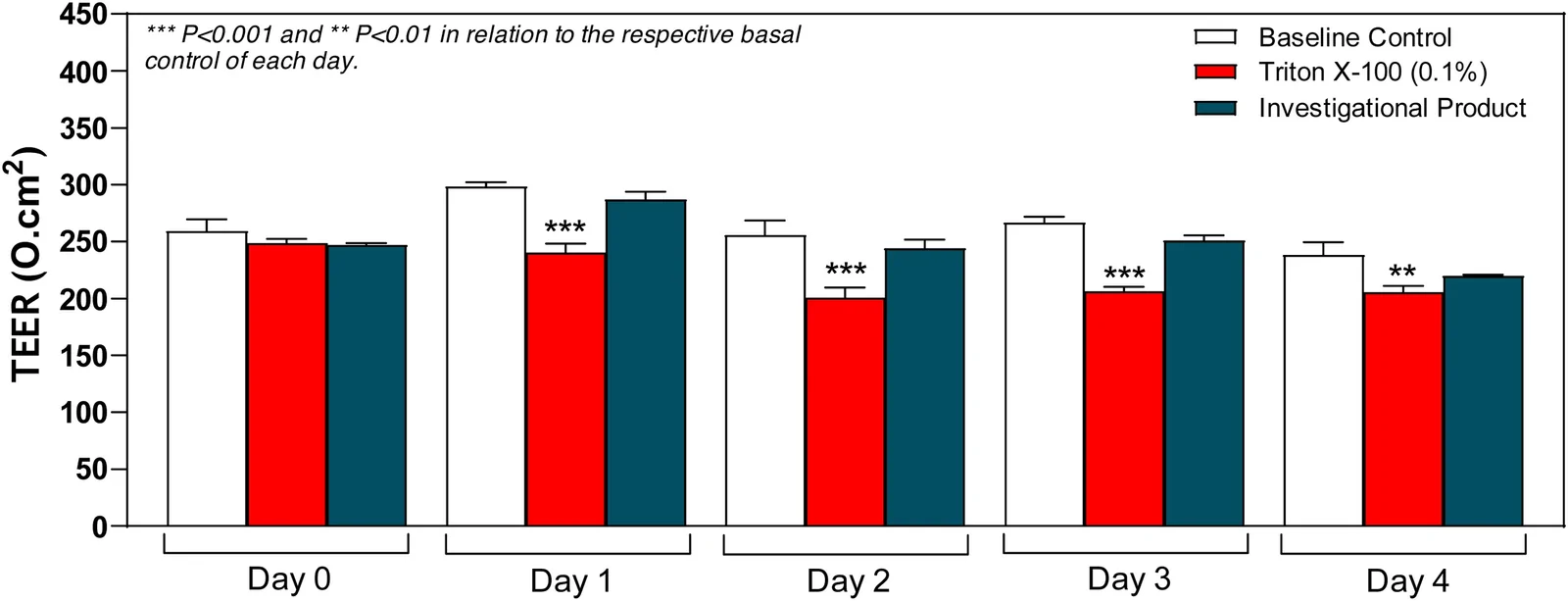
Transepithelial electrical resistance of reconstructed nasal epithelium treated with the investigational product. The epithelia were treated with IP, negative, and positive control during 4-days for subsequent TEER measurements. **p < 0.01 and ***p < 0.001 in relation to the respective negative control of each day.

**Fig. 3:**
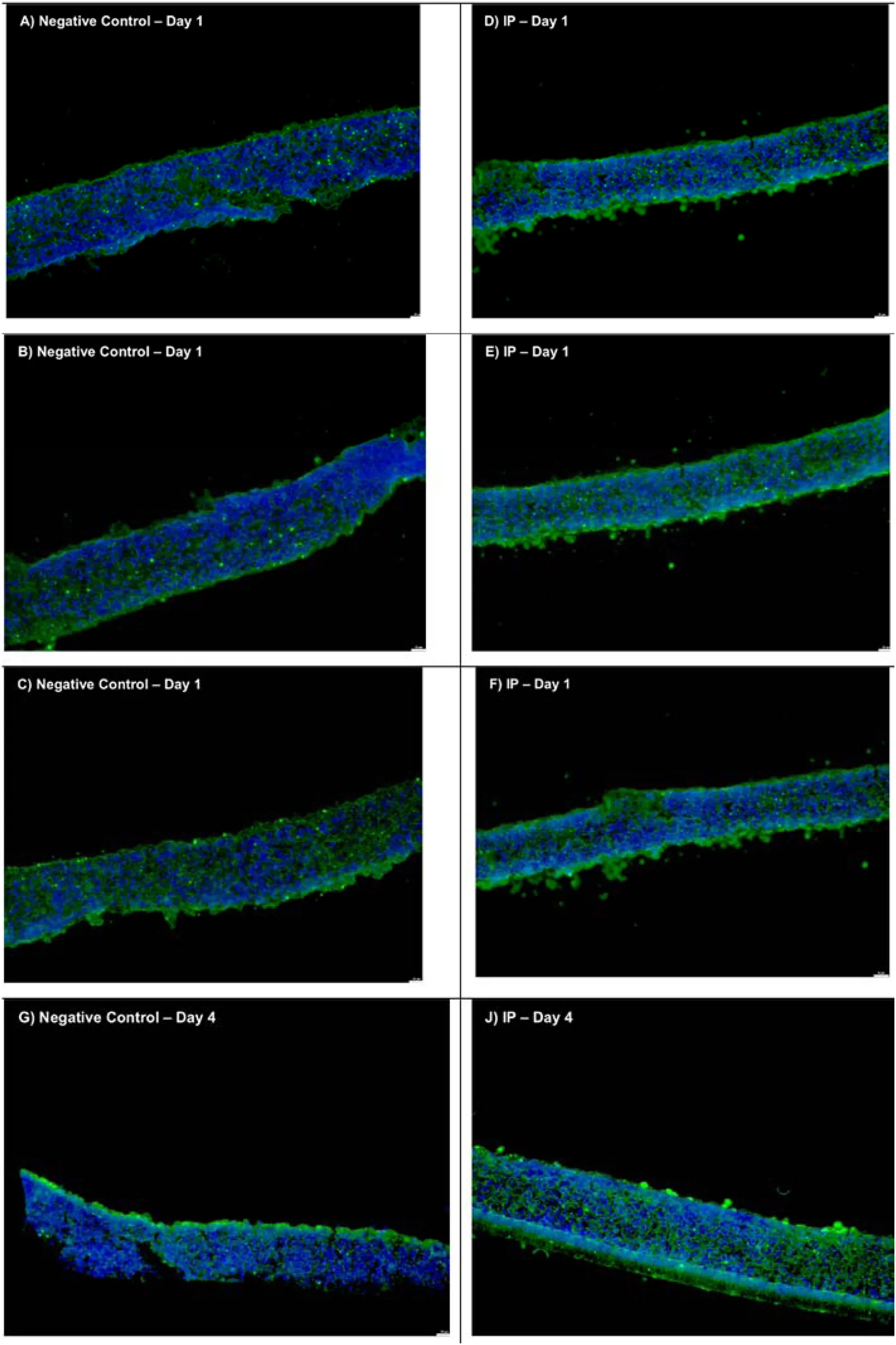
Representative images of each experimental group, showing imaging evaluation of AQP3 synthesis in the reconstructed nasal epithelium model treated with the IP over 4-days. The epithelia were treated with IP, negative, and positive control during 4-days for subsequent AQP-3 evaluation at days-1 and -4. The fluorescence emitted by the immunolabeling of Aquaporin-3 is shown in green and nuclear staining in blue. (A–C) Negative control ‒ Day-1; (D–F) Investigational product ‒ Day-1; (G–I) Negative Control ‒ Day-4; (J–L) Investigational product ‒ Day4.

The IP also maintained IL-1α and LDH (Figs. 2B and 3 C) synthesis values statistically similar to those observed in the respective control groups with saline solution, demonstrating that the investigational product does not induce an irritative and cytotoxic response, preserving cellular structures.

### TEER

The results demonstrated that exposure of the reconstructed nasal epithelia to the positive control (Triton X-100 ‒ 0.1%) promoted a statistically significant decrease in the Transepithelial Electrical Resistance (TEER) values, when compared to the respective negative controls, demonstrating disruption of the epithelium's integrity. On the other hand, treatment of the reconstructed nasal epithelia with the IP maintained the transepithelial electrical resistance values similar to those observed in the respective negative control group, on all days evaluated (Fig. 2). These results demonstrate that the investigational product can preserve the barrier structure of the nasal epithelium throughout days of experimentation.

### Aquaporin-3 expression

**Fig. 4:**
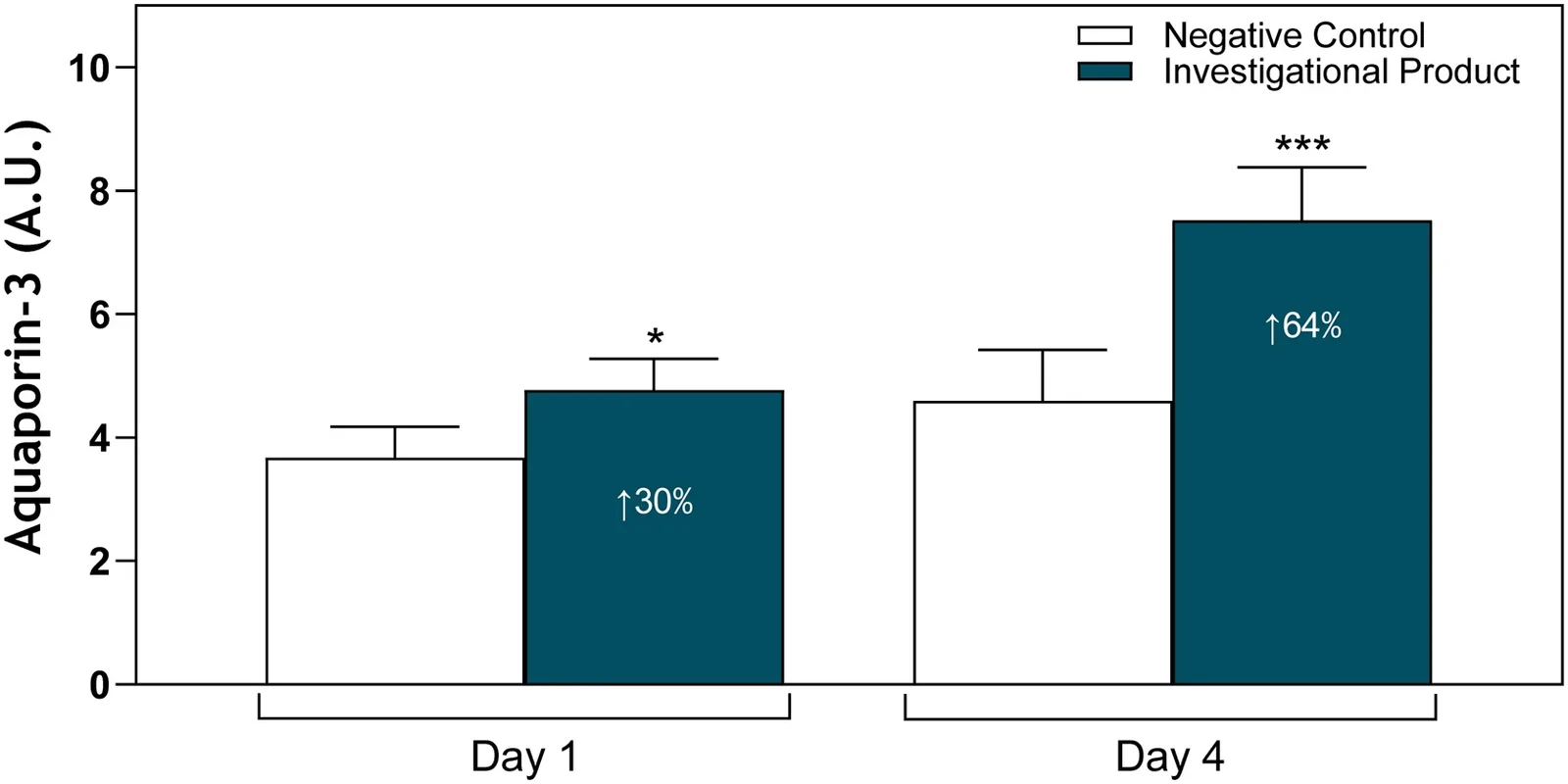
Semi-quantitative evaluation of AQP3 synthesis in the reconstructed nasal epithelium model treated with the IP over 4-days. The epithelia were treated with IP, negative, and positive control during 4-days for subsequent AQP-3 evaluation at days-1 and -4. * *p* < 0.05 and *** *p* < 0.001 in relation to the respective negative control of each day.

Treatment with the IP led to a 30% increase in AQP3 synthesis (*p* < 0.05) after 1-day of treatment and a 64% increase (*p* < 0.001) after 4-days of treatment, when compared to the respective negative control groups (saline solution), as shown in Fig. 3, Fig. 4.

### ATP quantification

**Fig. 5:**
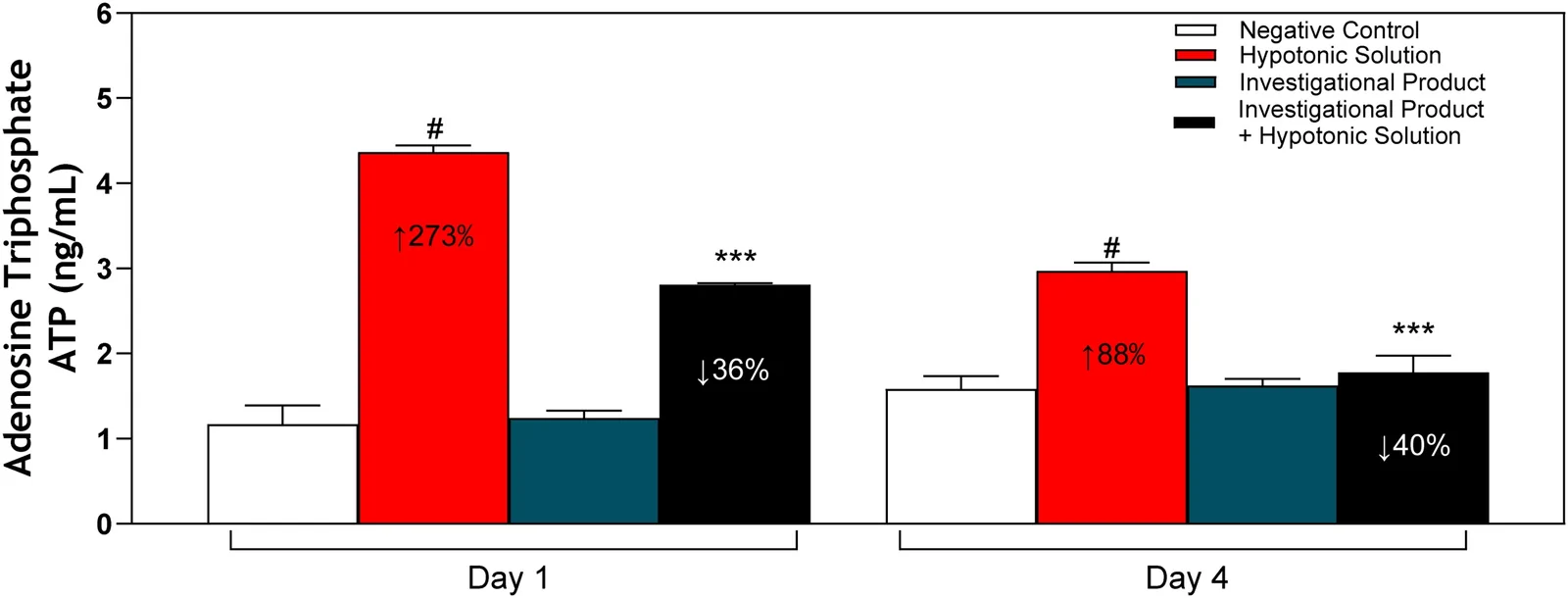
Quantitative evaluation of ATP synthesis in the reconstructed nasal epithelium model treated with the IP over 4-days. The epithelia were treated with IP, negative, and positive control during 4-days for subsequent ATP evaluation at days-1 and -4. # *p* < 0.001 in relation to the respective basal control of each day; *** *p* < 0.001 in relation to the respective Hypotonic Solution group for each day.

The exposure of the reconstructed epithelia to the positive control group (Hypotonic Solution) promoted a 273% (*p* < 0.001) increase in ATP release after 1-day of treatment and an 88% (*p* < 0.001) increase after 4 days of treatment, compared to the negative control group (saline solution), indicating the presence of a hypotonic stress environment. On the other hand, treatment with IP reduced ATP release by 36% (*p* < 0.001) on day-1 of treatment and 40% (*p* < 0.001) on day-4 of treatment, after the stress caused by hypotonic solution, demonstrating an ability to regulate water drainage in the nasal cavity. Additionally, treatment with IP alone did not induce ATP release when compared to the negative control group (Fig. 5).

### IL-8 synthesis

**Fig. 6:**
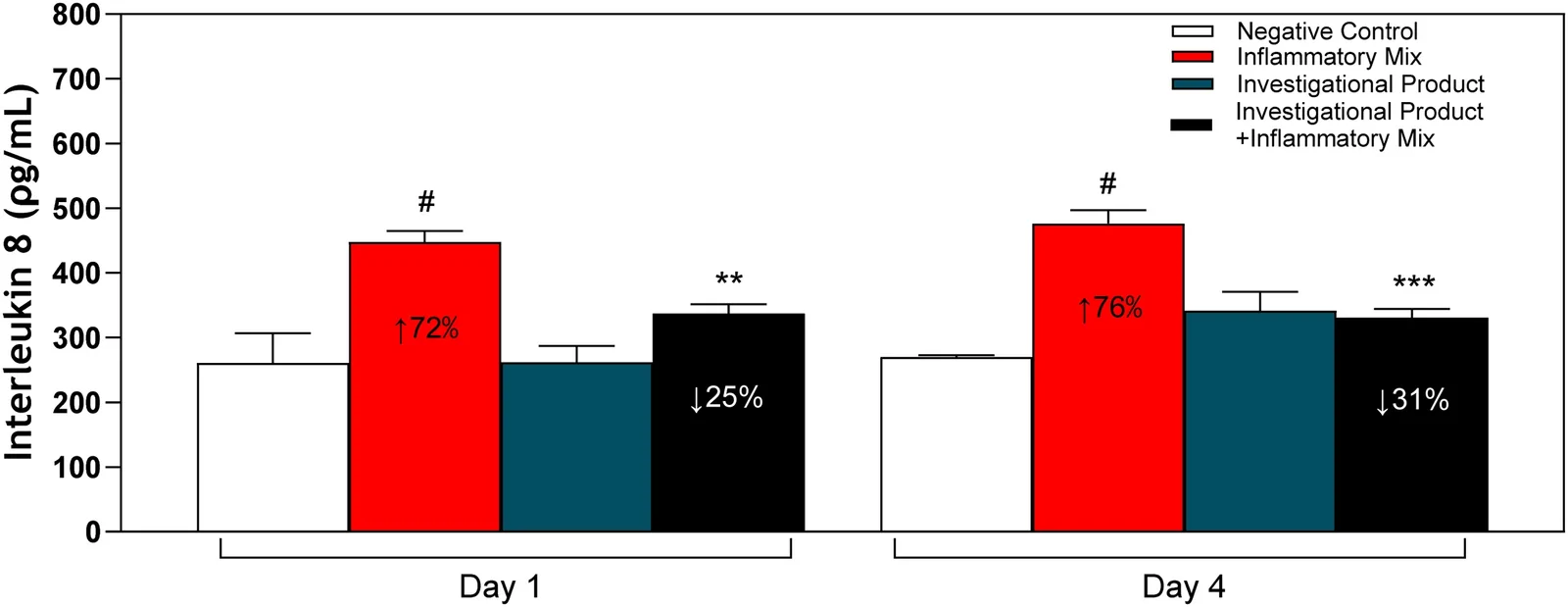
Quantitative evaluation of IL-8 synthesis in the reconstructed nasal epithelium model treated with the IP over 4-days. The epithelia were treated with IP, negative, and positive control during 4-days for subsequent IL-8 evaluation at days 1 and -4. # *p* < 0.001 in relation to the respective basal control of each day; # *p* < 0.001 in relation to the respective basal control of each day; ** *p* < 0.01 and *** *p* < 0.001 in relation to the Inflammatory Mix group.

The exposure of the reconstructed epithelia to the positive control group (inflammatory mix) promoted a 72% increase in IL-8 synthesis (*p* < 0.001) after 1-day of treatment and a 76% increase (*p* < 0.001) after 4-days of treatment, compared to the negative control group (saline solution), indicating activation of the inflammatory response. On the other hand, co-treatment of the inflammatory mix and the IP reduced IL-8 synthesis by 25% (*p* < 0.01) after 1-day of treatment and by 31% (*p* < 0.001) after 4-days of treatment, compared to the positive control group (inflammatory mix), demonstrating its ability to regulate the induced inflammatory response. Additionally, it was observed that treatment with the IP alone did not induce IL-8 synthesis when compared to the negative control group (Fig. 6).

## Discussion

Respiratory epithelium cultivated in Air-Liquid Interface (ALI) models are particularly useful for evaluating cellular and morphological reactivity to investigational products, as they closely mimic the structure and functions of human nasal tissue, including osmotic regulation and inflammatory responses. ALI culture allows for the differentiation of a well-structured epithelium that exhibits key morphological and functional features similar to the *in vivo* nasal mucosa, including the formation of tight junctions and the expression of specific epithelial proteins. These models also enable the assessment of inflammatory responses, such as the production of cytokines and chemokines, from the basolateral and/or apical surfaces, which are essential for understanding the cellular reactivity to external stimuli.^18^

Nasal mucosa serves as a protective barrier, preventing the entry of pathogens and irritants, while also playing a role in immune processes and air humidification.^19^ Therefore, products intended for the respiratory system should be able to maintain the cellular viability of the nasal epithelium. In conditions such as allergic and non-allergic rhinitis, acute and chronic rhinosinusitis, and postnasal drip, the use of saline solution is well established as it aids in mucus clearance, reduces nasal congestion, and supports the preservation of epithelial integrity.^20^ Importantly, saline solutions are known for their cellular safety, as they do not disrupt the viability or function of nasal epithelial cells, making them a suitable option for long-term or repeated use in both healthy and inflamed mucosa. Similarly, the IP results suggest that it is biocompatible and does not compromise epithelial integrity, supporting its safety for therapeutic applications.^21^^,^^22^

In contrast to positive control, our results demonstrated that the treatment with the IP preserved cell viability at levels comparable to the control groups treated with saline solution across all evaluated time points. These results indicate that the IP does not compromise cellular structures and lacks cytotoxic potential. Furthermore, the IP did not alter the synthesis levels of IL-1α or LDH, which remained statistically similar to those of the saline-treated control groups. This indicates that the IP does not induce an irritative or inflammatory response in the nasal epithelium and further supports its non-aggressive profile toward epithelial cells. In addition to maintaining cellular viability and a non-irritative profile, the IP preserved the integrity of the epithelial barrier. While exposure to the positive control led to a significant reduction in TEER, indicative of epithelial disruption, treatment with the IP maintained TEER values comparable to baseline controls throughout the study period. This demonstrates the IP's ability to maintain epithelial barrier function and structural integrity.

Homeostasis in nasal mucosa physiology involves hydration and preservation of osmotic regulation. In this scenario, aquaporins are a family of 13 different small membrane proteins that act as selective barriers to the transport of water and small solutes through a dynamic electrostatic attraction. Among them, Aquaporin-3 (AQP-3) is highly expressed in the nasal mucosa upon exposure to water loss, suggesting that this marker protein provides water to the cells, protecting them from dehydration and maintaining the epithelial microenvironment, preserving osmotic conditions.^23^^,^^24^

The increase in Aquaporin-3 (AQP3) expression, induced by the IP in the reconstructed epithelium, refers to the elevated levels of this membrane transport protein, which facilitates the movement of water and glycerol across cell membranes. AQP3 plays a crucial role in regulating fluid transport and maintaining cellular homeostasis in various parts of the body, including the upper and lower airways. Modulation of AQP3 expression may be relevant in respiratory conditions such as rhinitis and sinusitis, as the product demonstrates potential in promoting cellular hydration and preserving osmotic balance, although it has maintained the same mucociliary clearance potential as shown by the saline solution.^25^

Extracellular Adenosine Triphosphate (ATP) is an important luminal paracrine signal that regulates the hydration of the surface of human airway epithelial cultures through its action on apical membrane purinergic receptors. Airway epithelial cells release ATP as a paracrine regulator in response to stress situations, such as hypotonic stress-induced swelling and loss of cell membrane integrity.^26^^,^^27^ IP treatment, during hypotonic stress, reduced ATP release, which suggests that IP may have a protective hydrosmotic control balance on the extracellular environment, or eventually a direct modulatory effect on the epithelial cell response to stress, possibly influencing the regulation of airway surface hydration or fluid drainage.

IL-8 is an inflammatory chemokine known for attracting neutrophils to sites of inflammation, playing a key role in respiratory conditions. The literature suggests that reducing IL-8 levels may be associated with decreased inflammation and related symptoms. For example, Turner et al. demonstrated that IL-8 concentration in nasal secretions correlated with the severity of symptoms in rhinovirus-induced colds, indicating that lower IL-8 levels could be associated with milder symptoms.^28^ Therefore, the investigational product’s ability to reduce IL-8 synthesis suggests its anti-inflammatory potential and capacity to modulate the inflammatory response.

These findings, taken together, highlight the favorable IP safety profile by preserving epithelial viability, barrier integrity, and exhibiting no cytotoxic or pro-inflammatory effects. Its ability to modulate hydration-related markers, such as AQP3 and ATP, alongside the reduction of IL-8 synthesis, supports its potential role as a safe and promising agent for osmotic regulation and inflammation control in the nasal epithelium. Further studies, including clinical trials, are recommended to validate its efficacy and establish its applicability in nasal health management.

## Conclusion

Our study demonstrates that the Investigational Product (IP) with minerals and xylitol is safe and has potential benefits for nasal epithelial health, including control of hydration and osmotic balance. Additionally, the product exhibited anti-inflammatory properties by reducing IL-8 synthesis, suggesting a potential role in inflammatory conditions such as in rhinitis and rhinosinusitis.

## ORCID IDs

Michelle Sabrina da Silva: 0000-0002-9319-2384

Douglas Costa Morais: 0000-0001-5160-2501

Flávia Cardoso Males: 0000-0002-2026-1836

Mariana Rodrigues Pinto: 0000-0002-8840-4805

Stevin Zung: 0000-0002-8172-0928

Edwin Tamashiro: 0000-0002-3153-6292

## Funding

This work was supported by Aché Laboratórios Farmacêuticos S.A.

## Data availability statement

The authors declare that all data are available in repository.

## Declaration of competing interest

Dr. Michele Silva is executive director of MEDCIN VITRO and received research funding from Aché Laboratórios Farmacêuticos S.A. Dr. Edwin Tamashiro is a medical consultant for Aché Laboratórios Farmacêuticos S.A. All other authors are employees of Aché Laboratórios Farmacêuticos S.A.
